# Magnetic capsulate triboelectric nanogenerators

**DOI:** 10.1038/s41598-021-04100-2

**Published:** 2022-01-07

**Authors:** Pengcheng Jiao, Ali Matin Nazar, King-James Idala Egbe, Kaveh Barri, Amir H. Alavi

**Affiliations:** 1 Institute of Port, Coastal and Offshore Engineering, Ocean College, Zhejiang University, Zhoushan, 316021 Zhejiang China; 2 Hainan Institute of Zhejiang University, Sanya, 572025 Hainan China; 3 Engineering Research Center of Oceanic Sensing Technology and Equipment, Zhejiang University, Ministry of Education, Hangzhou, China; 4 Department of Civil and Environmental Engineering, University of Pittsburgh, Pittsburgh, PA USA; 5 Department of Computer Science and Information Engineering, Asia University, Taichung, Taiwan

**Keywords:** Devices for energy harvesting, Civil engineering

## Abstract

Triboelectric nanogenerators have received significant research attention in recent years. Structural design plays a critical role in improving the energy harvesting performance of triboelectric nanogenerators. Here, we develop the magnetic capsulate triboelectric nanogenerators (MC-TENG) for energy harvesting under undesirable mechanical excitations. The capsulate TENG are designed to be driven by an oscillation-triggered magnetic force in a holding frame to generate electrical power due to the principle of the freestanding triboelectrification. Experimental and numerical studies are conducted to investigate the electrical performance of MC-TENG under cyclic loading in three energy harvesting modes. The results indicate that the energy harvesting performance of the MC-TENG is significantly affected by the structure of the capsulate TENG. The copper MC-TENG systems are found to be the most effective design that generates the maximum mode of the voltage range is 4 V in the closed-circuit with the resistance of 10 GΩ. The proposed MC-TENG concept provides an effective method to harvest electrical energy from low-frequency and low-amplitude oscillations such as ocean wave.

## Introduction

Triboelectric nanogenerators (TENG) are energy harvesting systems capable of generating electrical power using the triboelectric effect. They have marked their debut a decade ago^1^ and have been attracting significant attention ever since then^2^. Given the critical energy crisis and severely ecological deterioration worldwide due to the consumption of conventional fossil fuels, it is important to reshape the irreproducible mineral resources-based conventional energy structure and develop environment-friendly, renewable green energy technologies such as TENG^3^. As a novel sustainable energy solution, TENG have been developed to generate electrical power from the continuous, independent, easily accessible, and widespread mechanical energy^4^. Advances in TENG technology has led to various energy harvesting applications, including self-powered biomedical devices (e.g., electronic skins^5,6^, mechnosensational communication system^7^, and wearable systems^8^), self-power engineering devices (e.g., temperature fluctuation^9^, and structural vibration^10,11^), and various sensing devices (e.g., motion vector sensing^12,13^, wearable sensors^14,15^, driving monitoring^16^, fish bladder film-based position monitoring^17^, self-functional healthcare and monitoring socks^18^, and human–machine interfacing^19^). Research effort has been dedicated to improving the performance of the TENG-enabled devices^20^. Since TENG are typically designed by tribo-materials in simple structures, structural design plays a dominant role in the energy harvesting performance of these nanogenerators^21,22^. For example, studies were carried out on the TENG-based prototypes designed in the spring-assisted multi-layered structures^23^, robust swing structures^24^, water balloon structures^25^, whirling-folded structures^26,27^, floating oscillator-embedded structures^28–30^, etc. Architected structural materials (e.g., mechanical metamaterials) have been deployed to improve the performance of nanogenerators^31^. More recently, studies have been carried out on applying computer-aided techniques in TENG, e.g., TENG-enabled internet of things^32^, or artificial intelligence-enhanced TENG^33^. In general, it is critical and challenging to rationally design simple structures that are feasible in commercial production to effectively trigger TENG under undesirable mechanical excitations in the environment (e.g., low-frequency and low-amplitude oscillations)^2,34^.

Here, we develop the magnetic capsulate TENG (MC-TENG) for energy harvesting under undesirable external excitations. TENG are designed in the capsulate structure, which are placed in a holding frame between driven magnets. A dielectric capsulate TENG is driven by the oscillation-triggered magnetic force into a holding electrode frame to generate electrical power in a freestanding triboelectric layer mode. Experiments are conducted on the capsulate TENG designed in three modes to investigate the influences of the metal materials (i.e., copper and aluminum), number of the magnets, and dielectric-to-electrode connection strategy on the energy harvesting performance of the MC-TENG. The energy harvesting performance of the MC-TENG is significantly affected by the structure of the capsulate TENG. There are certain motion parameters to maximize the power output of TENG from the perspectives of the structural design (e.g., decreasing layer thickness, increasing surface area, or obtaining conformal contact and complete separation between layers), the material selection (e.g., increasing the triboelectric charge density and reducing the dielectric constant), and the excitation maneuverability (e.g., enhancing frequency or improving amplitude). Comparing to the existing structures on energy harvesting in ocean, the proposed MC-TENG are developed under the considerations of fatigue and possible loss of elasticity. The magnetic configuration in this study utilizes magnets to replace the lever arms^35^ or springs^36,37^ such that to reduce the fatigue of the materials. In particular, magnets are used to provide the transmitting force from the ocean waves to the TENG capsules in the loading phase, and enable the return of the capsules to their original positions in the unloading phase. As a result, the MC-TENG are observed with well durability over a long time period. Taking advantage of the magnetic capsulate structure, the MC-TENG systems can harvest electrical energy under low-frequency and low-amplitude oscillations. The rest of the paper is drawn as: “Results and discussion” section presents the design principle, experimental setup, fabrication, and testing results of the MC-TENG. “Conclusions” section summarizes the main findings on the MC-TENG. “Materials and methods” section indicates the materials and methods used in this study.

## Results and discussion

### Design principle of MC-TENG

**Figure 1:**
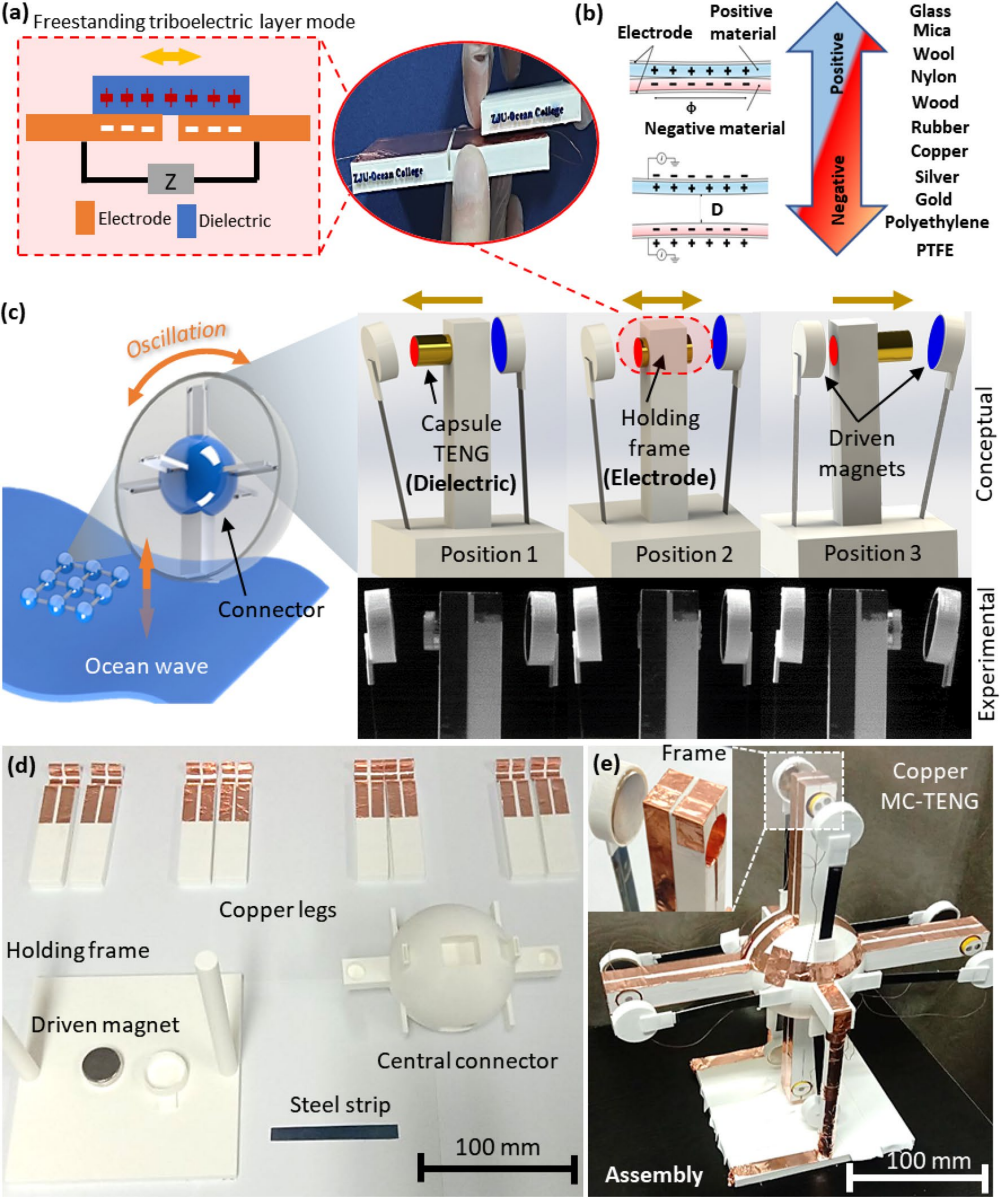
Design principle of the MC-TENG. (**a**) Principal illustration of the freestanding triboelectric layer mode used in the MC-TENG. (**b**) series of triboelectric material^40^. (**c**) Application of the MC-TENG in ocean wave, the three oscillation scenarios of the capsulate TENG (i.e., the dielectric) in the holding frame (i.e., the electrode) between the driven magnets, and the process comparison with the experimental observations recorded by a high-speed camera (conceptual images are created by SolidWorks 2017 SP4.1 https://www.solidworks.com/). (**d**) The components of the copper MC-TENG including the copper legs, holding frame, central connector, steel strips, and driven magnets. (**e**) Design details and assembly of the copper MC-TENG with four magnetic capsulate systems.

The MC-TENG comprises of the dielectric capsulate TENG, the conductive holding frame, and the driven magnets. Figure 1 demonstrates the design principle of an MC-TENG. Figure 1a conceptually illustrates the freestanding triboelectric layer mode used in the nanogenerators and Fig. 1b demonstrates the series of possible triboelectric materials^38–40^. Figure 1c illustrates the application of the MC-TENG under low-frequency and low-amplitude oscillation in ocean wave. The oscillation leads to the initial displacement of the capsulate TENG in the holding frame. Since the capsules are embedded with the end magnets that are oppositive of the driven magnets, the magnetic force pushes/attracts the capsules to periodically move in the holding frame. The oscillation scenarios of the capsulate TENG in the holding frame between the driven magnets are conceptualized, which are compared with the experimental observations recorded by a high-speed camera (see Supplemental Video 1). Figure 1d displays the components of the copper MC-TENG, including the copper legs, holding frame, central connector, steel strips, and driven magnets. The components were fabricated using the 3D printing technique using the PLA material. The elastic steel strips are particularly used to enhance the sliding motion of the capsulate TENG. Since the MC-TENG is developed based on the freestanding triboelectric layer mode, it is critical to enhance the sliding motion of the capsulate TENG between the driven magnets. The driven magnets are particularly designed with the elastic steel strips due to two reasons: (1) The driven magnets are able to move through much longer distance between the driven magnets since the steel strips are deflected by the oscillations. (2) The steel strips are deflected by the oscillations, which converts the external mechanical energy into the elastic energy of the deformed steel strips. This is significant for the reported MC-TENG systems, especially considering the fact that low-frequency and low-amplitude oscillations are typically not stable in reality. Figure 1e presents the assembly of the copper MC-TENG assembled by four capsulate TENG. Electrical power is generated from the oscillated motions of the copper capsules. The oscillation response and energy harvesting performance of the four-capsule, copper MC-TENG are demonstrated in Supplemental Video 2. The material and geometric properties of the capsulate TENG are provided in Supplemental Information Sec. 1.

### Experimental setup and fabrication of the MC-TENG

In this study, the MC-TENG were fabricated and tested with respect to the structure and material of the capsulate TENG. In particular, the capsules were designed in the structures with single-layered (mode 1), double-layered (mode 2) and non-layered (mode 3) copper or aluminum films. The numbers of end magnets in the capsulate TENG were varied from 1 to 8 on each end of the capsules (i.e., 1 to 4 magnets on 1 or 2 layers). Two layers of copper or aluminum are coated on the inside of the hole in the holding frame, which are located with the distance gap of 1 mm from each other (see Fig. 1d). The main difference between mode 1, mode 2 and mode 3 is the connection strategy of the capsulate TENG to the holding frame. In particular, mode 1 was coated by one copper or aluminum layer and then covered by a Kapton layer. The copper or aluminum layer of the capsulate TENG was not connected to the copper or aluminum layers on the hole in the holding frame. Mode 2, on the contrary, was coated by two separate copper or aluminum layers that were spaced 1 mm and then covered by a Kapton layer. Each copper or aluminum layer of the capsulate TENG was connected to the copper or aluminum layers on the hole in the holding frame. Mode 3, however, was only coated by a Kapton layer that was not connected to the holding frame. Output voltage is generated when electrons are transferred from the metal layers on the capsulate TENG (i.e., dielectric) to the holding frame (i.e., i.e., electrode).

**Figure 2:**
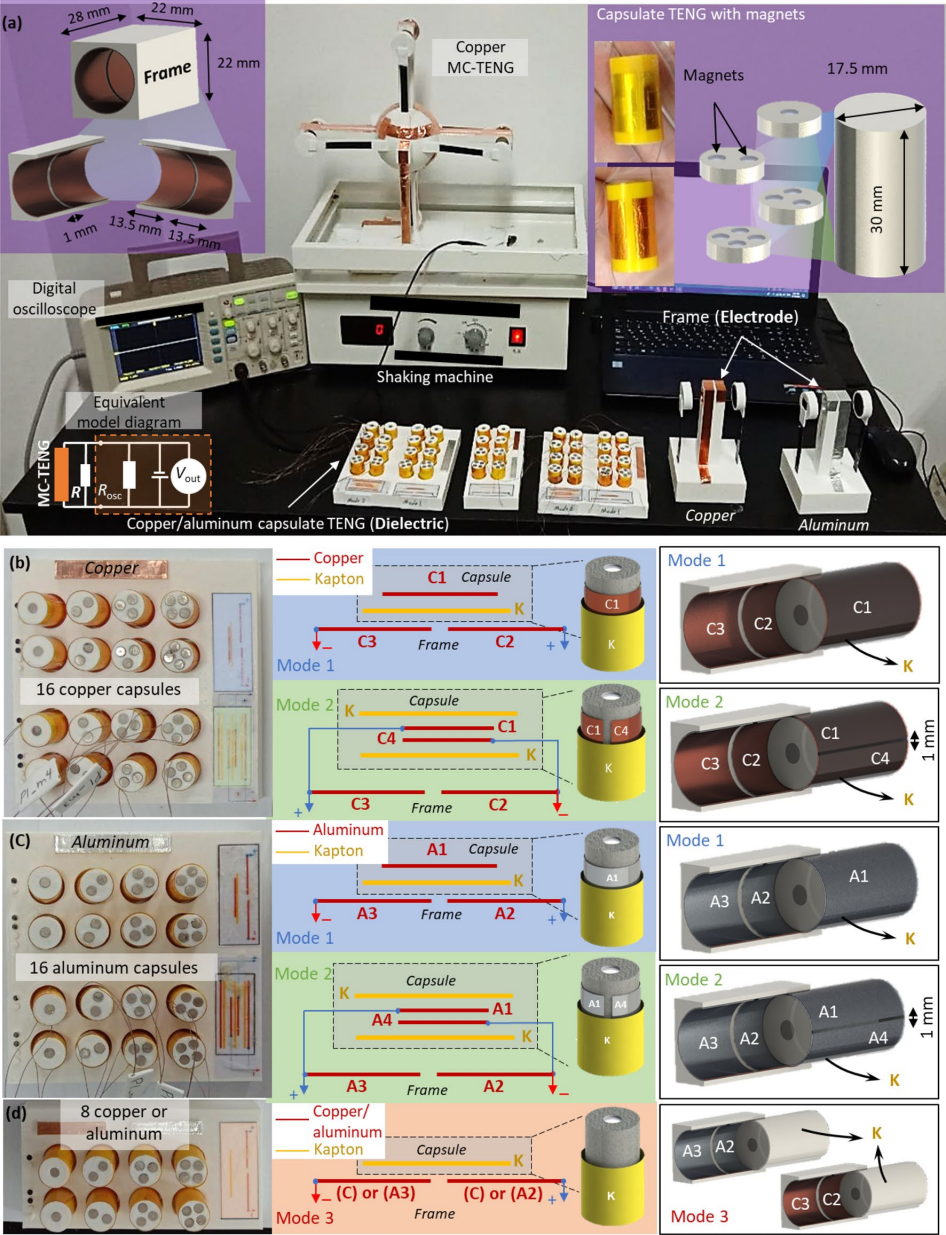
Fabrication of the capsulate TENG and experimental setup of the MC-TENG. (**a**) Experimental setup of the MC-TENG fabricated with the materials of copper and aluminum in the single-layered (mode 1), double-layered (mode 2) and non-layered (mode 3) structures. Design and fabrication of the capsulate TENG using (**b**) copper and (**c**) aluminum in mode 1 and mode 2 with different end magnets. (**d**) Design and fabrication of the copper/aluminum capsulate TENG in mode 3 with different end magnets (all the capsules were fabricated with 1 to 4 end magnets on 1 or 2 layers) (Conceptual images are created by SolidWorks 2017 SP4.1 https://www.solidworks.com/).

Figure 2a shows the equivalent model diagram and the experimental setup of the copper or aluminum MC-TENG embedded with the end magnets. The shaking machine was applied to create the oscillation motion with the constant speed of 220 rpm, and the digital oscilloscope was used to collect the voltage signal. The equivalent impedance of the oscilloscope was the resistance of 1 MΩ, which was in parallel to the capacitor of 15 pF. The oscilloscope was connected in parallel to the energy harvesting circuit. Larger output voltage was observed with larger resistors while smaller voltage was measured under smaller resistance. In addition, when the resistors were equal to or higher than the impedance of the oscilloscope, the obtained voltage was almost the same as the open-circuit voltage although no external resistor was connected. Figure 2b presents the capsulate TENG in copper. The copper strips were designed in the single-layered structure in mode 1 and double-layered structure in mode 2. The external copper layers (i.e., C2 and C3 in mode 1, and C1 and C4 in mode 2) were attached to the holding frame, which were connected to the open or closed-circuit. The Kapton layer was used between different copper layers. The capsulate TENG and holding frame were not connected in mode 1 and connected in mode 2. Figure 2c presents the aluminum capsules in the MC-TENG. The structures of the MC-TENG (i.e., modes 1 and 2) and the number of the end magnets were maintained the same as the copper capsulate TENG. Figure 2d displays the capsulate TENG in mode 3. i.e., the capsules designed without the copper or aluminum layer. In particular, the capsules in mode 3 were covered by the Kapton layer and the copper or aluminum layers were only attached to the holding frame. Note that the MC-TENG were fabricated via 3D printing at a millimeter scale. However, the nanogenerators can create electrical power at the micro/nanoscale. As long as the primary characteristics are maintained, the MC-TENG systems can be efficiently triggered at the multiscale. In order to scale down the MC-TENG systems to the micro/nanoscale, it is significant to maintain the design principle (i.e., the freestanding triboelectric layer mode) and the geometric ratios (e.g., the ratio between the capsulate TENG length and its distance to the driven magnets).

### Voltage of the copper and aluminum MC-TENG in the three modes

**Figure 3:**
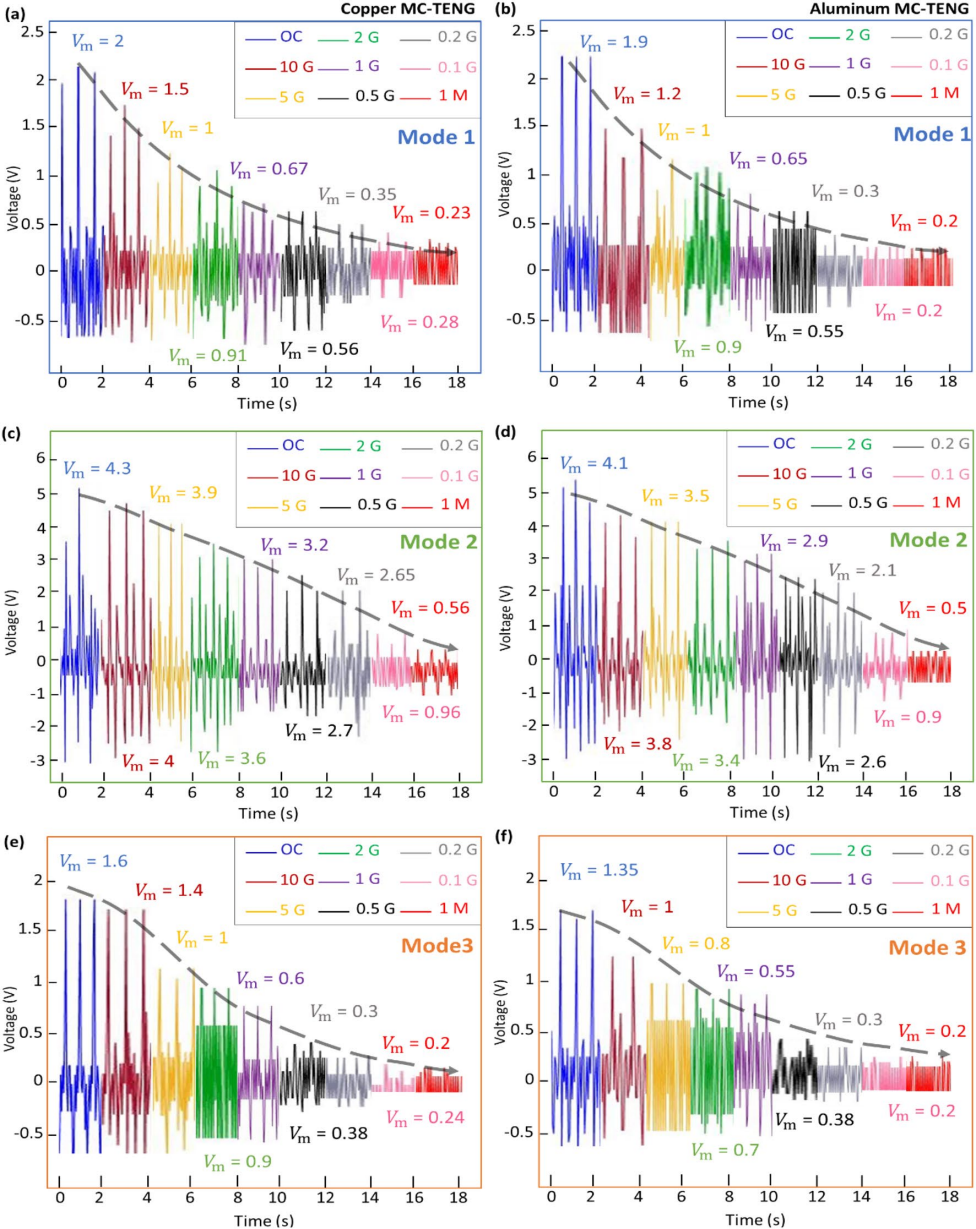
Energy harvesting performance of the copper and aluminum MC-TENG in the three modes. Voltage distribution trends of the (**a**) copper and (**b**) aluminum MC-TENG in mode 1, (**c**) copper and (**d**) aluminum MC-TENG in mode 2, and (**e**) copper and (**f**) aluminum MC-TENG in mode 3 (all the capsulate TENG are designed with 4 end magnets on each end, the loading time is fixed as 2 s, and the electrical resistance in the closed-circuit is varied from 1 MΩ to 10 GΩ).

Figure 3 investigates the energy harvesting performance of the copper or aluminum MC-TENG consisted of one capsulate TENG. The capsules are designed with single layer of copper or aluminum in mode 1, double layer in mode 2, and non-layer in mode 3. All the capsules are designed with 4 end magnets on each end. The loading time is fixed as 2 s for each case and the electrical resistance in the closed-circuit is varied from 1 MΩ to 10 GΩ. Figure 3a presents the output voltage of the copper MC-TENG in mode 1 in the open and closed-circuit. The maximum mode of the voltage range is 4 V that is obtained in the open circuit. Higher voltages are observed when the resistance is larger and the minimum output is ${\mathrm{V}}_{\mathrm{min}}=0.28\,{\mathrm{V}}$. Figure 3b shows the output voltages of the aluminum MC-TENG in mode 1. Similar voltage distribution trend is obtained with respect to the electrical resistance. Figure 3c,d compare the voltages of the copper and aluminum MC-TENG in mode 2. The maximum voltages are obtained in the open circuit and the minimum voltages are in the closed-circuit with the smallest electrical resistance of 1 MΩ. In the same manner as mode 1 and mode 2, Fig. 3e,f compare the voltages of the copper and aluminum MC-TENG in mode 3. Similar voltage distribution trends are observed in terms of the electrical resistance. According to the material comparison between the copper and aluminum MC-TENG, it is observed that the voltage of the aluminum capsules is slightly smaller than that of the copper ones, since the electrical resistance of copper is smaller than aluminum. According to the comparison of the structures between the MC-TENG in mode 1, mode 2 and mode 3, it is measured that the capsulate TENG in mode 2 generates the highest voltage while mode 3 is the lowest design. The highest voltage is approximately three times higher than the lowest, which is because more copper or aluminum layers are designed in mode 2, i.e., mode 2 creates the largest triboelectric surface area for electrons to transfer comparing with mode 1 and mode 3. In addition, the metal layers of the capsulate TENG in mode 2 are connected to the metal layers in the holding frame. Comparing with mode 1, however, the lack of the metal layer in mode 3 does not significantly affect the energy harvesting performance of the MC-TENG.

### Influences of the end magnets, material and structure on the MC-TENG

**Figure 4:**
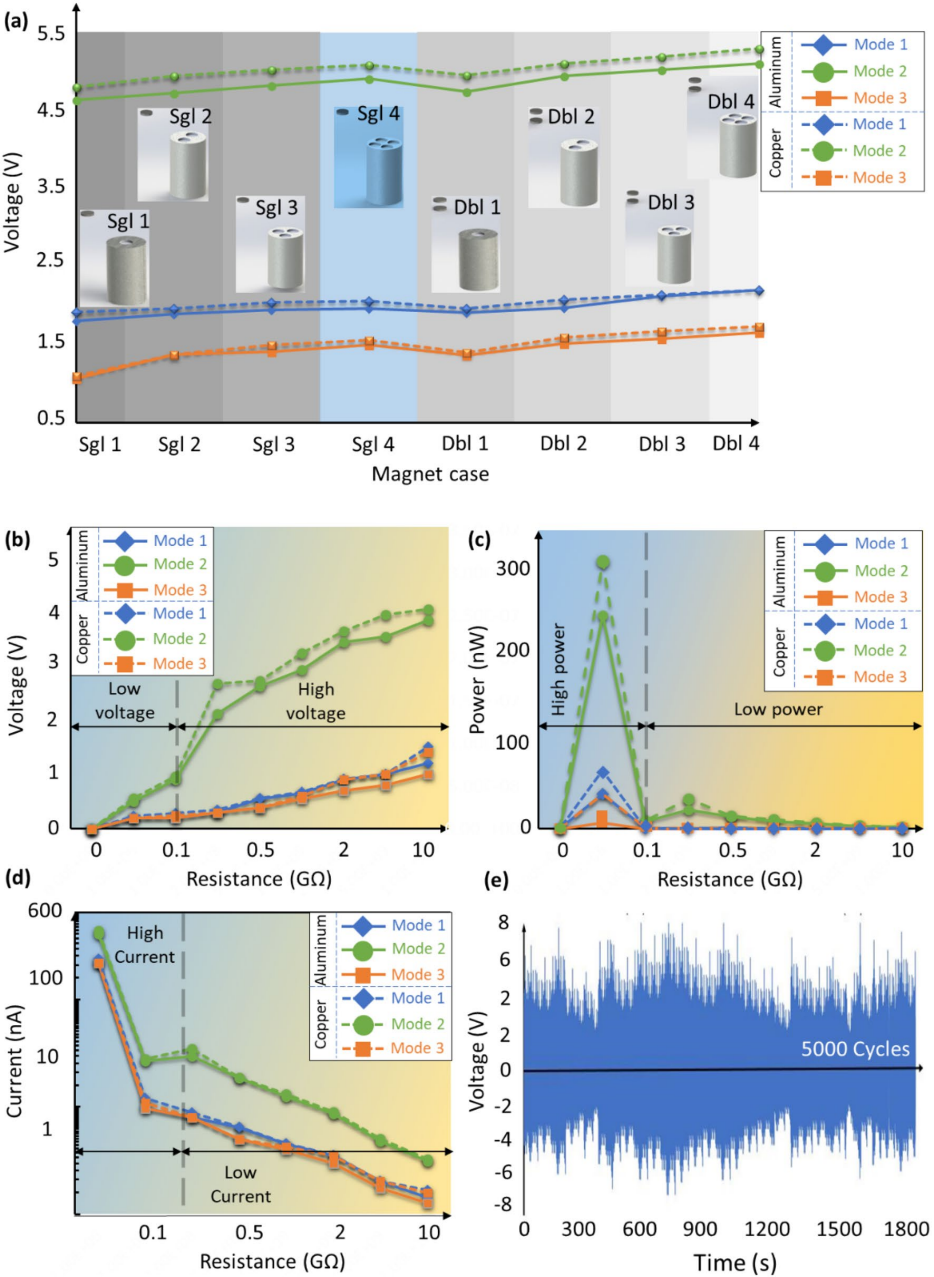
Influences of the end magnets, material and structure on the energy harvesting performance of the MC-TENG. (**a**) Voltage distribution with respect to the end magnets for the copper or aluminum MC-TENG in the open-circuit. (**b**) Voltage (**c**) power and (**d**) current distributions of the MC-TENG in terms of the electrical resistance in the closed-circuit with 4 end magnets on each end (Sgl 4) (all the capsulate TENG are designed in mode 1, mode 2 and mode 3). (**e**) Stability test of MC-TENG under 5000 cyclic loading during 1800s.

The energy harvesting response of the MC-TENG is studied with respect to the end magnets, material and structure. The output voltage and electrical power are investigated for the copper or aluminum MC-TENG in mode 1, mode 2 and mode 3 with different numbers of end magnets. The end magnets of the capsulate TENG are particularly changed from 1 magnet (Sgl 1) to 8 magnets (Dbl 4). Figure 4 displays the comparison of the obtained voltage and power between the copper and aluminum MC-TENG in the closed-circuit. Figure 4a compares the voltage distributions of the MC-TENG in terms of the number of the end magnets in the closed-circuit with the electrical resistance of 10 GΩ. In can be seen that the voltage of the MC-TENG is not significantly affected by the number of the end magnets or the material of the capsules for all the three modes. On the contrary, the structure of the capsulate TENG critically affects the energy harvesting performance, i.e., the output voltage of the capsulate TENG designed in the double-layered structure in mode 2 is higher than two times of the capsules in mode 1 and approximately three times of the capsules in mode 3. Referring to the principle of the freestanding triboelectric layer mode, the electrical performance of MC-TENG is significantly affected by the sliding motion between the capsulate TENG and the holding frame. As a consequence, the structure of the capsulate TENG plays a critical role in the energy harvesting performance. On the contrary, the output voltage of the copper MC-TENG is slightly better than that of the aluminum MC-TENG because of its better conductivity property. In addition, number of the end magnets does not significantly affect the MC-TENG electrical performance because the sliding motion of the capsulate is mainly determined by the deflection of the steel strips. In other words, the sliding distance of the capsulate TENG between the driven magnets is mainly dependant on by the bending deformation of the steel strips. Figure 4b demonstrates the voltage of the copper and aluminum MC-TENG with 4 end magnets in the three modes with respect to the electrical resistance. The MC-TENG in mode 2 offer the highest voltage, especially for the closed-circuit with the electrical resistance higher than 0.1 Ω. Only slightly difference is obtained between the copper and aluminum MC-TENG in mode 1 and mode 3. Figure 4c shows the obtained power of the MC-TENG with the electrical resistance. According to the findings, it is concluded that the material of copper and the structure of mode 2 are the most effective designs for the MC-TENG. The peak power of nearly 400 nW is obtained under the electrical resistance of $50\,{\mathrm{M}}\Omega$.

The output voltage and current of the MC-TENG are affected by the electrical properties of the materials. Based on Ohm’s law, this input impedance results in a significant voltage drop at the output. Ideally, in a situation with an input impedance, there wouldn’t be a voltage drop. In general, the material properties and dimensions of TENG layers have a significant impact on the output behavior of TENG. Herein, the effect of major device parameters is evaluated. Triboelectric charge density (${\upsigma }_{\mathrm{T}}$) is affected by the relative position of triboelectric pairs in the triboelectric series, triboelectric surface structuring, contact area influenced by applied force, and environmental factors. Although in MC-TENG did not measure the charge density (${\upsigma }_{\mathrm{T}}$) during the experiment, using the copper and making suitable triboelectric surfaces for collecting the peak power have been considered. The power output increases with increasing ${\upsigma }_{\mathrm{T}}$. The environmental factors such as humidity, pressure, temperature, and surrounding medium have been shown to affect triboelectric charge density and, therefore, could result in variations in the steady state ${\upsigma }_{\mathrm{T}}$ value^41^. Since the same environmental factors used to collect the voltages in MC-TENG, the effects are constant across all modes. The thickness of the constituent TENG layers has a direct impact on electric field propagation and polarization, and thus on power output. In MC-TENG mode 2, the length (L) of the TENG has increased. The effect of TENG layer size was investigated by varying the length (L) of the TENG. Increasing L increases the surface area of TENG layers, which raises the peak output power^41^.

### Performance prediction in numerical simulations

**Figure 5:**
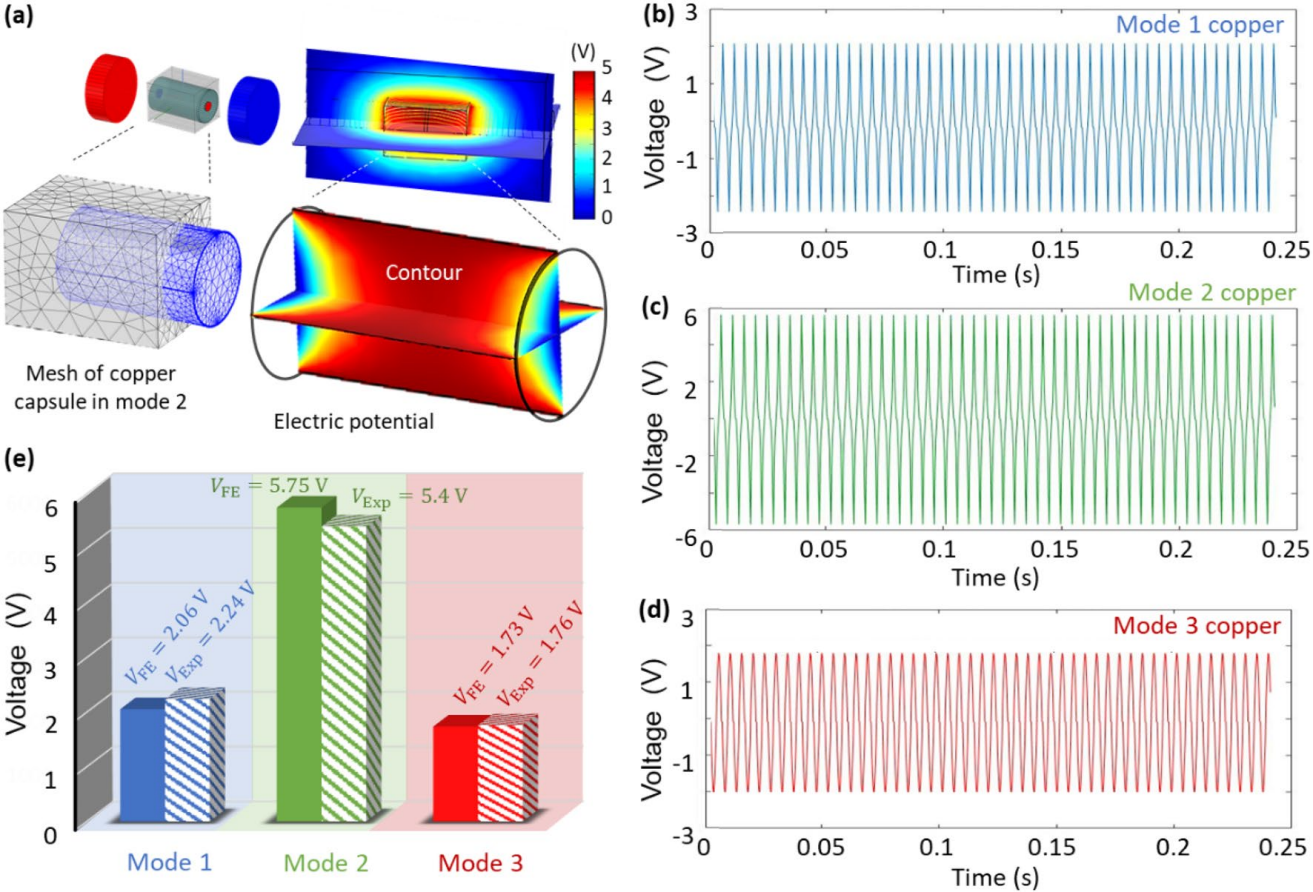
Numerical modeling of the copper MC-TENG and comparing with the experiments. (**a**) Numerical setup, mesh and electrical potential contour of the copper capsule in mode 2. Output voltages of the copper MC-TENG in (**b**) mode 1, (**c**) mode 2, and (**d**) mode 3. (**e**) Comparison of the voltage between the experimental results (${\mathrm{V}}_{\mathrm{Exp}}$) and numerical results (${\mathrm{V}}_{\mathrm{FE}}$) for the copper MC-TENG (all the capsules are designed with 1 end magnet on each end, and the voltages are obtained in the closed-circuit with the electrical resistance of 10 GΩ).

Numerical models are developed to validate the energy harvesting performance of the MC-TENG. According to the experimental findings, the capsulate TENG are designed by the material of copper and 1 end magnet on each end. Figure 5 displays the numerical modelling and results obtained in COMSOL and Ansys Maxwell (Supplemental Information Sec. 1). Figure 5a shows the numerical setup, mesh and electrical potential contour for the copper capsulate TENG in mode 2 in the closed-circuit with the resistance of 10 GΩ. Figure 5b,c,d presents the output voltages of the copper MC-TENG with the capsules designed in mode 1, mode 2 and mode 3, respectively. Figure 5e compares the experimental and numerical voltages of the copper MC-TENG in the three modes. Satisfactory agreements are obtained with the maximum difference of ${\mathrm{Diff}}_{\mathrm{max}}=6.1{\%}$ in Mode 2.

## Conclusions

In this study, we developed the magnetic capsulate triboelectric nanogenerators (MC-TENG) for energy harvesting under undesirable mechanical excitations (e.g., low-frequency and low-amplitude oscillations). The dielectric TENG were designed in the capsulate structure, which were investigated with respect to the structure (i.e., single-layered in mode 1, double-layered in mode 2, and non-layered in mode 3), the material (i.e., copper and aluminum), and the number of end magnets. Experiments were conducted and the findings indicated that the energy harvesting performance of the MC-TENG was significantly affected by the structure of the capsulate TENG. The copper MC-TENG systems in mode 2 were found the most effective designs with the maximum mode of the voltage range is 4 V in the closed-circuit and a resistance of 10 GΩ. Based on the magnetic capsulate structural design of the sustainable nanogenerators, the reported MC-TENG systems are capable of converting small-scale mechanical motions into electrical energy. Further research can be conducted to demonstrate the capability of charging small electronic devices (e.g., sensors deployed in remote offshore locations) under low-frequency and low-amplitude oscillations such as ocean wave.

## Materials and methods

### Fabrication of the MC-TENG

In this study, the copper legs, holding frame, and central connector were 3D printed by the material of PLA (Polymaker-Tough) using the 3D printer (Ultimaker-S3, Ultimaker Inc.) with the maximum printing size of 223 $\times$ 223 $\times$ 205 mm and the maximum printing velocity of 24 mm/s.

### Experimental setup and measurement

In the experiments, the low-frequency and low-amplitude oscillations was created by the shaking machine (Digital Oscillator HY-4A). The output voltage was measured using the digital oscilloscope (RIGOL DS1102E, RIGOL Tech. Inc.). The high-speed camera Motion pro Y7-s3 (Integrated Design Tools, IDT Inc.) with the maximum speed of 10,600 FPS was used to record the oscillation scenarios of the capsulate TENG in the MC-TENG (see Supplemental Information Sec. 2). The contact surfaces of the copper or aluminum layers between the capsules and holding frame were clearly wiped and the lubrication was applied to reduce the friction.

### Numerical simulations

The 3D finite element models were developed using COMSOL and Ansys Maxwell. The energy harvesting performance of the copper MC-TENG was investigated using COMSOL Multiphysics, and the effect of the end magnets on the energy harvesting efficiency of the capsulate TENG was studied in Ansys Maxwell. The linear quadrilateral shell elements (S4R) were considered in the numerical study. Details of the numerical models are provided in Supplemental Information Sec. 3.

## Supplementary Information

Supplementary Information 1.

Supplementary Video 1.

Supplementary Video 2.
