# Supplementary material for: Magnetic capsulate triboelectric nanogenerators
Source: Sci Rep. 2022 Jan 7;12:89. doi: 10.1038/s41598-021-04100-2 (PMC8741797; doi:10.1038/s41598-021-04100-2)
Supplement: Supplementary file 1 — Supplementary Information 1. [file 41598_2021_4100_MOESM1_ESM.docx]

Supplemental Information for

**Magnetic Capsulate Triboelectric Nanogenerators**

Pengcheng Jiao^a,b,1^, Ali Matin Nazar^a^, King-James Idala Egbe^a^, Kaveh Barri^c^, Amir H. Alavi^c^

*^a^: Institute of Port, Coastal and Offshore Engineering, Ocean College, Zhejiang University, Zhoushan 316021, Zhejiang, China ( Corresponding author. Email:* [*pjiao@zju.edu.cn*](mailto:pjiao@zju.edu.cn) *)*

*^b^: Engineering Research Center of Oceanic Sensing Technology and Equipment, Zhejiang University, Ministry of Education, China*

*^c^: Department of Civil and Environmental Engineering, University of Pittsburgh, Pittsburgh, PA, USA*

**1. Material and Geometric Properties of the Capsulate TENG in the Experiments**

The material and geometric properties of the capsulate TENG in the experiments are provided in Table S1.

**Table S1.** Material and geometric properties of the material and geometric properties in the capsulate TENG.

|  | Component | Parameter | Value |
| --- | --- | --- | --- |
| Material properties | Copper | Resistance (Ohm$\cdot$mm^2^/m) | 0.0171 |
|  |  | Conductivity (S$\cdot$m/ mm^2^) | 58.5 |
|  | Aluminum | Resistance (Ohm$\cdot$mm^2^/m) | 0.0283 |
|  |  | Conductivity (S$\cdot$m/ mm^2^) | 61 |
|  | Kapton | Length (mm) | 54.95 |
|  |  | Width (mm) | 30 |
|  |  | Thickness (mm) | 0.11 |
|  | Magnets | Permeability | 1.1 |
|  |  | Conductivity (Siemens/m) | $625000$ |
|  |  | Magnitude (A/m) | -890000 |
| Geometric properties  (mm) | Overall capsules | Length | 30 |
|  |  | Diameter | 17.5 |
|  |  | Maximum gap bw.  capsule and frame | 1 |
|  | Driven magnets | Diameter | 30 |
|  |  | Thickness | 10 |
|  | End magnets | Diameter | 5 |
|  |  | Thickness | 1 |

**2. Experimental Setup**

Fig. S1 displays the devices used in the experiments. Fig. S1(a) shows the shaking machine used to apply the oscillation to the copper MC-TENG, Fig. S1(b) shows the digital oscilloscope used to measure the output voltage, and Fig. S1(c) shows the high-speed camera used to record the oscillation scenarios of the capsulate TENG in the MC-TENG.

**
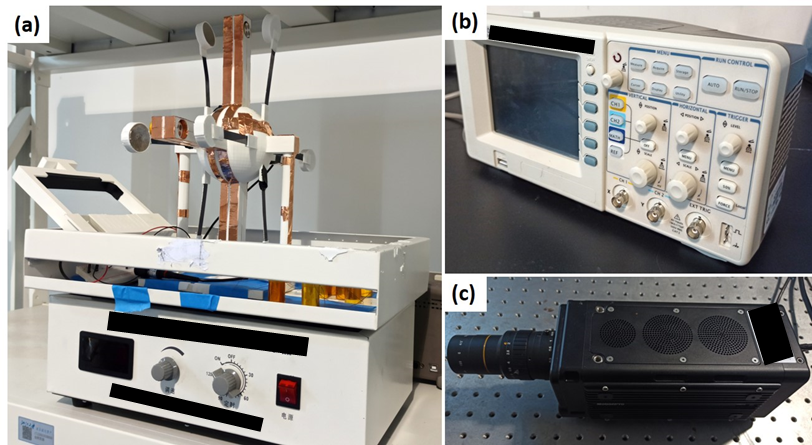
**

**Fig. S1.** (a) The copper MC-TENG on the shaking machine (Digital Oscillator HY-4A). (b) The digital oscilloscope used to measure the output voltage (RIGOL DS1102E). (c) High-speed camera (Integrated Design Tools) used to record the oscillation scenarios of the capsulate TENG in the MC-TENG.

**3. Numerical Simulations**

Numerical models were developed to investigate the energy harvesting performance of the copper MC-TENG in mode 2 using COMSOL Multiphysics, and the effect of the end magnets on the energy harvesting efficiency of the capsulate TENG using Ansys Maxwell software.


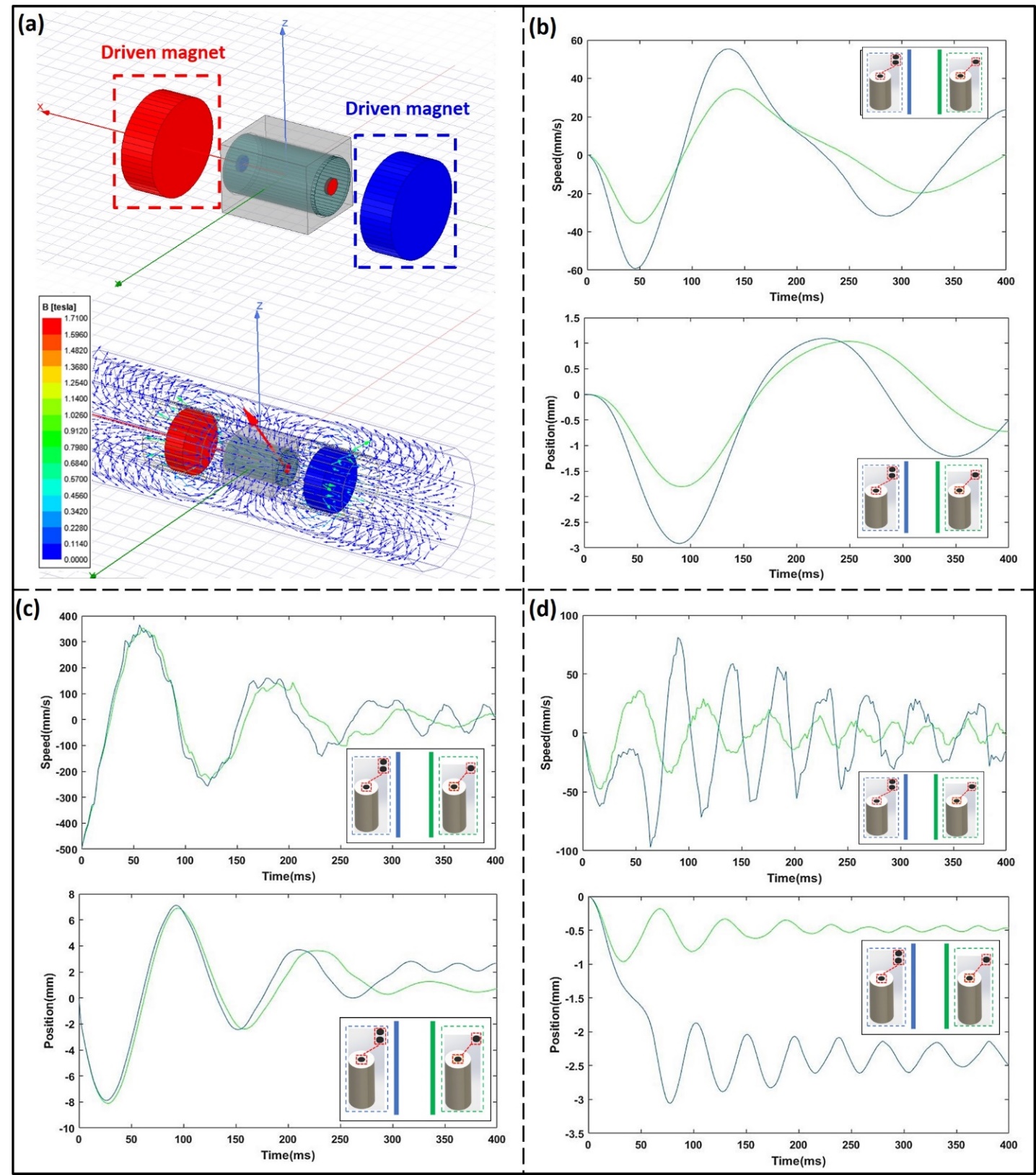


**Fig. S2.** (a) Magnetic field and mesh structure. (b) Speed and position of the capsule TENG with 1 and 2 end magnets on each end. (c) Position and speed of the first driven magnet (red) with an initial velocity of 500 mm/s. (d) Position and speed of the second driven magnet (blue).

**3.1 Effect of the End Magnets on Energy Harvesting Efficiency of Capsulate TENG**

Ansys Maxwell was used to study the influence of the end magnets on the energy harvesting efficiency of the capsule TENG. The mesh structure and magnetic field are shown in Fig. S2. The linear quadrilateral shell elements of type S4R were considered in the numerical study. The numerical simulations were conducted using Neodynium magnets with the same size and properties as the experiments (Table S1). In this study, we particularly compared the velocity rate of two capsule states. In the first case, the capsule contains 1 end magnet on each end was examined, as shown in Fig. S2(a), while the capsule in the second case contains 2 end magnets on each side. An initial velocity of 500 mm/s was applied to the end magnet with the damping of 1 N$\cdot$s/m. The maximum velocity of the capsulate TENG was 37.5 mm/s in the first case, and the maximum velocity for the second case was 60 mm/s. The finite element (FE) simulations indicate that increasing the number of the end magnets enhances the velocity of the capsulate TENG.

**3.2 Energy Harvesting Performance of the Copper MC-TENG in the Three Modes**

The energy harvesting performance of the copper MC-TENG was simulated in COMSOL Multiphysics. The mesh structure of the capsulate TENG was defined as the moving mesh with $dx=14\sin(2\pi t)$ and $dy=dz=0$, as shown in Fig. S3. The tribo-charge surface density was $\sigma=4.2\times{10}^{-7} Cm^{-2}$ and the velocity was 220 rmp.


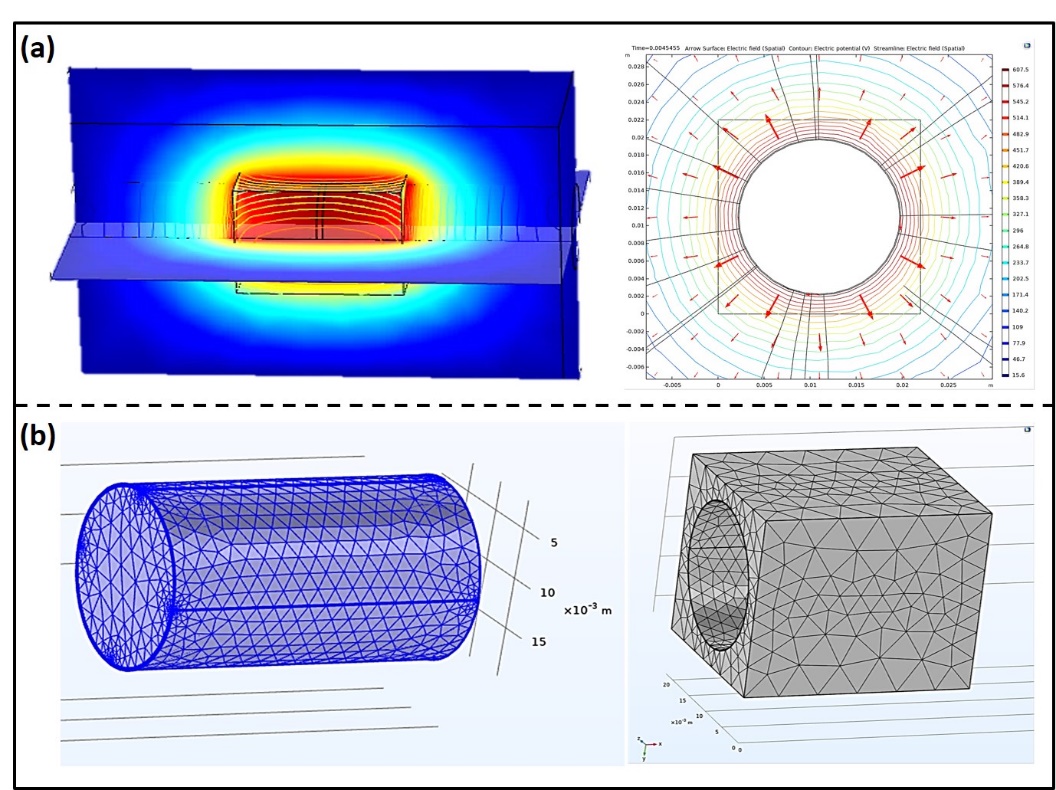


**Fig. S3.** (a) Distribution of electrical potential in the passage path for the copper capsulate TENG. (b) Mesh structure of the capsule TENG and rectangular cube cavity.

**4.** **Voltage of the Copper and Aluminum MC-TENG in the Three Modes**

Fig. S4-S9 investigates the energy harvesting signals of the copper or aluminum MC-TENG (all the capsulate TENG are designed with 4 end magnets on each end, the loading time is fixed as 12 s, and the electrical resistance in the closed-circuit is varied from 1 MΩ to 10 GΩ).


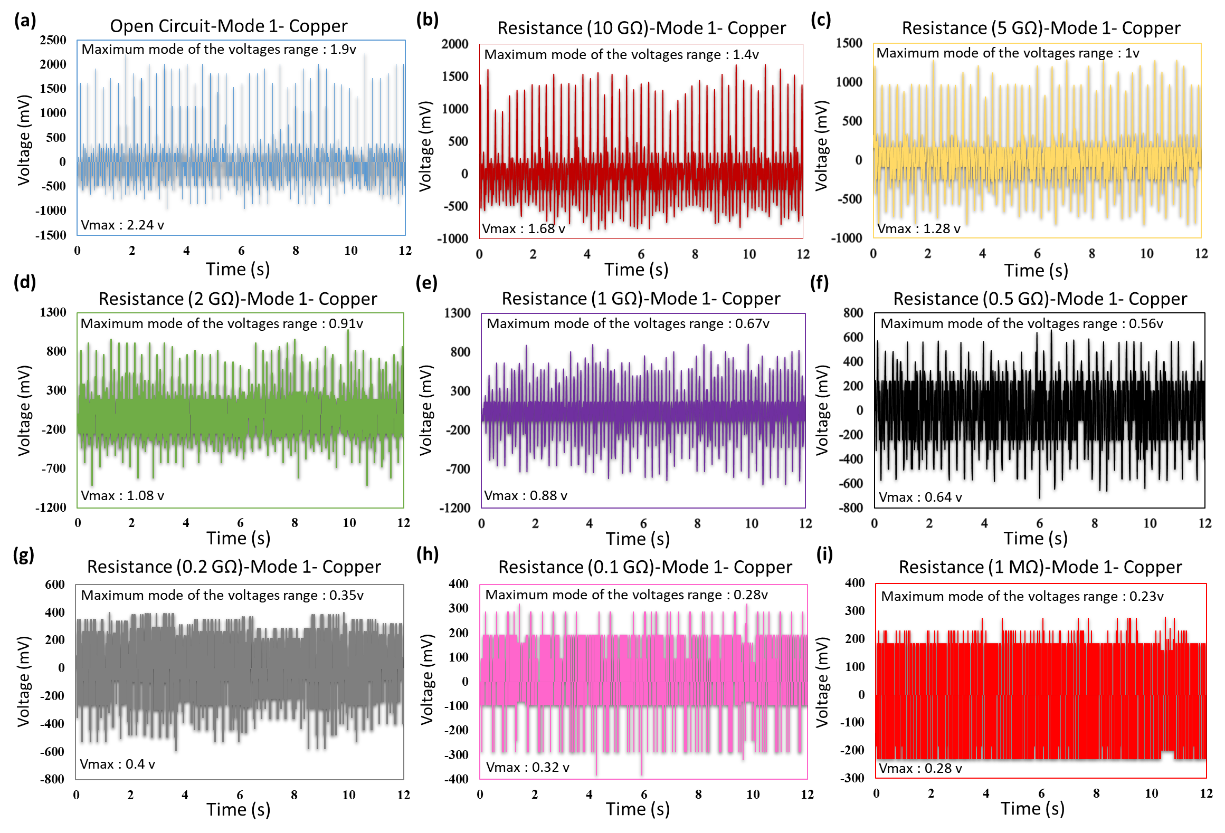


**Fig. S4.** **Energy harvesting signals of the copper MC-TENG in Mode 1.** (a) Voltage distribution of copper in open circuit. (b) Voltage distribution of copper with the electrical resistance 10ΩG, (c) 5 ΩG, (d) 2 ΩG, (e) 1 ΩG, (f) 0.5 ΩG, (g) 0.2 ΩG, (h) 0.1 ΩG, (i) 1MG.


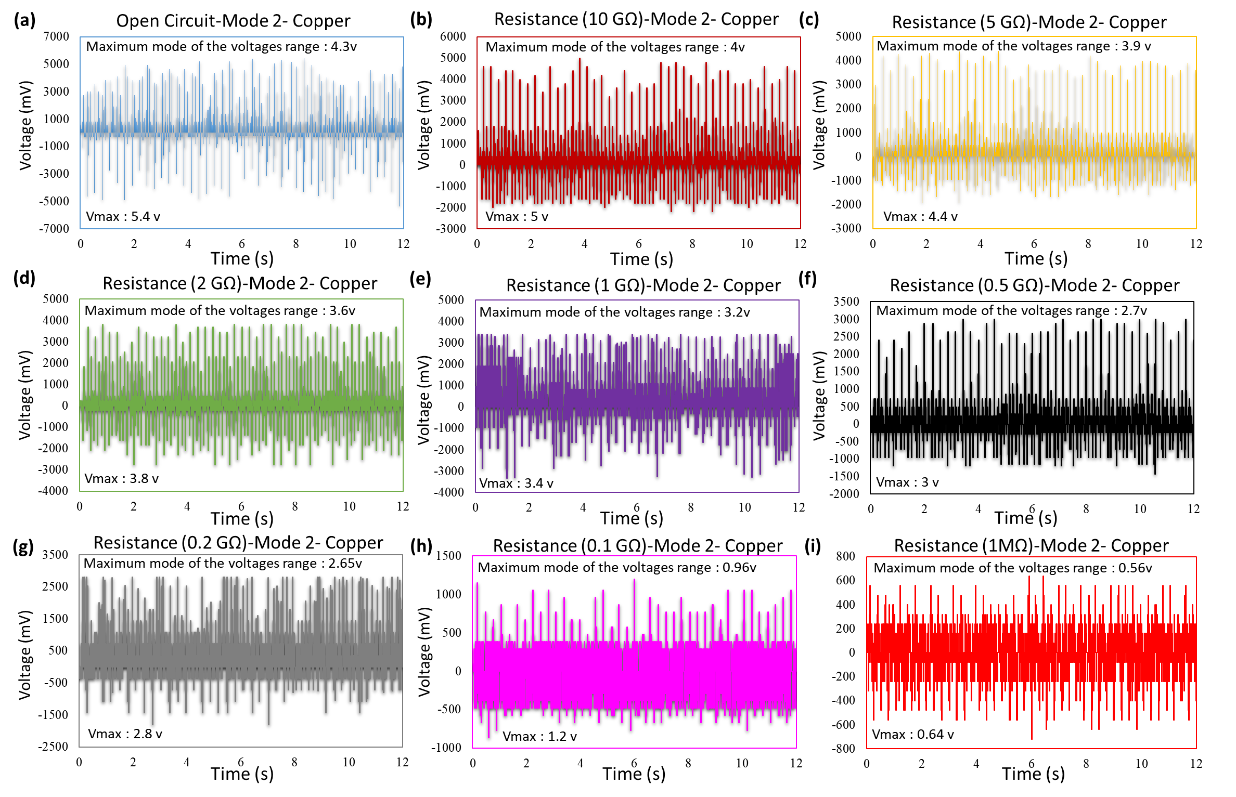


**Fig. S5.** **Energy harvesting signals of the copper MC-TENG in Mode 2.** (a) Voltage distribution of copper in open circuit. (b) Voltage distribution of copper with the electrical resistance 10ΩG, (c) 5 ΩG, (d) 2 ΩG, (e) 1 ΩG, (f) 0.5 ΩG, (g) 0.2 ΩG, (h) 0.1 ΩG, (i) 1MG.


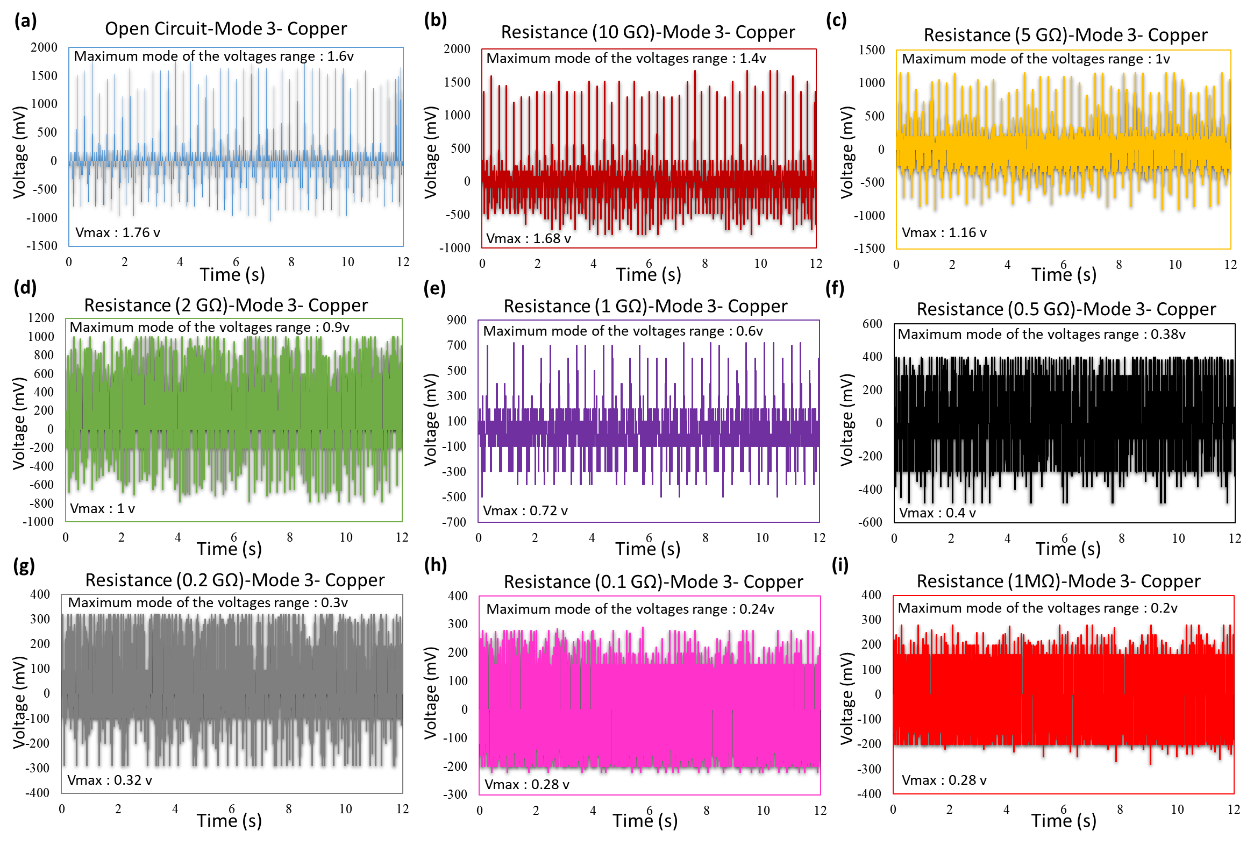


**Fig. S6.** **Energy harvesting signals of the copper MC-TENG in Mode 3.** (a) Voltage distribution of copper in open circuit. (b) Voltage distribution of copper with the electrical resistance 10ΩG, (c) 5 ΩG, (d) 2 ΩG, (e) 1 ΩG, (f) 0.5 ΩG, (g) 0.2 ΩG, (h) 0.1 ΩG, (i) 1MG.


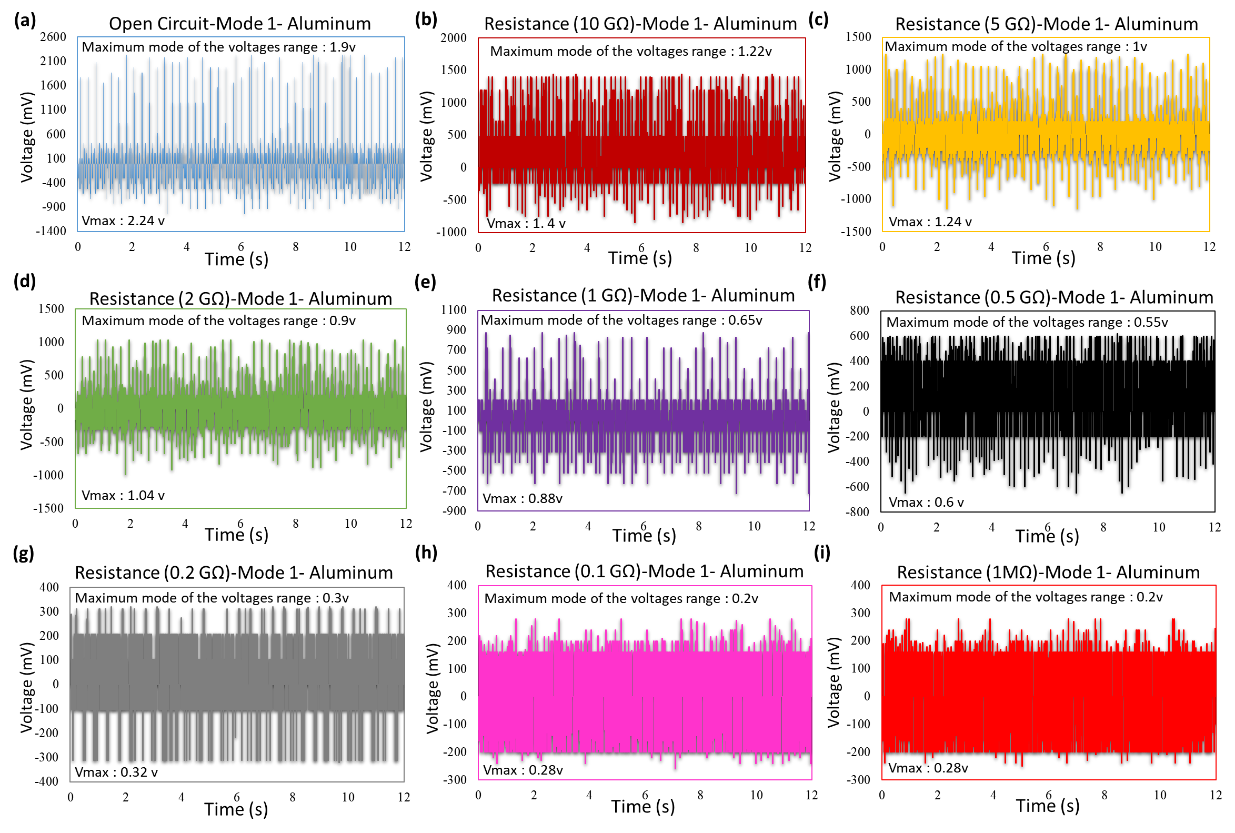


**Fig. S7.** **Energy harvesting signals of the Aluminum MC-TENG in Mode 1.** (a) Voltage distribution of copper in open circuit. (b) Voltage distribution of copper with the electrical resistance 10ΩG, (c) 5 ΩG, (d) 2 ΩG, (e) 1 ΩG, (f) 0.5 ΩG, (g) 0.2 ΩG, (h) 0.1 ΩG, (i) 1MG.


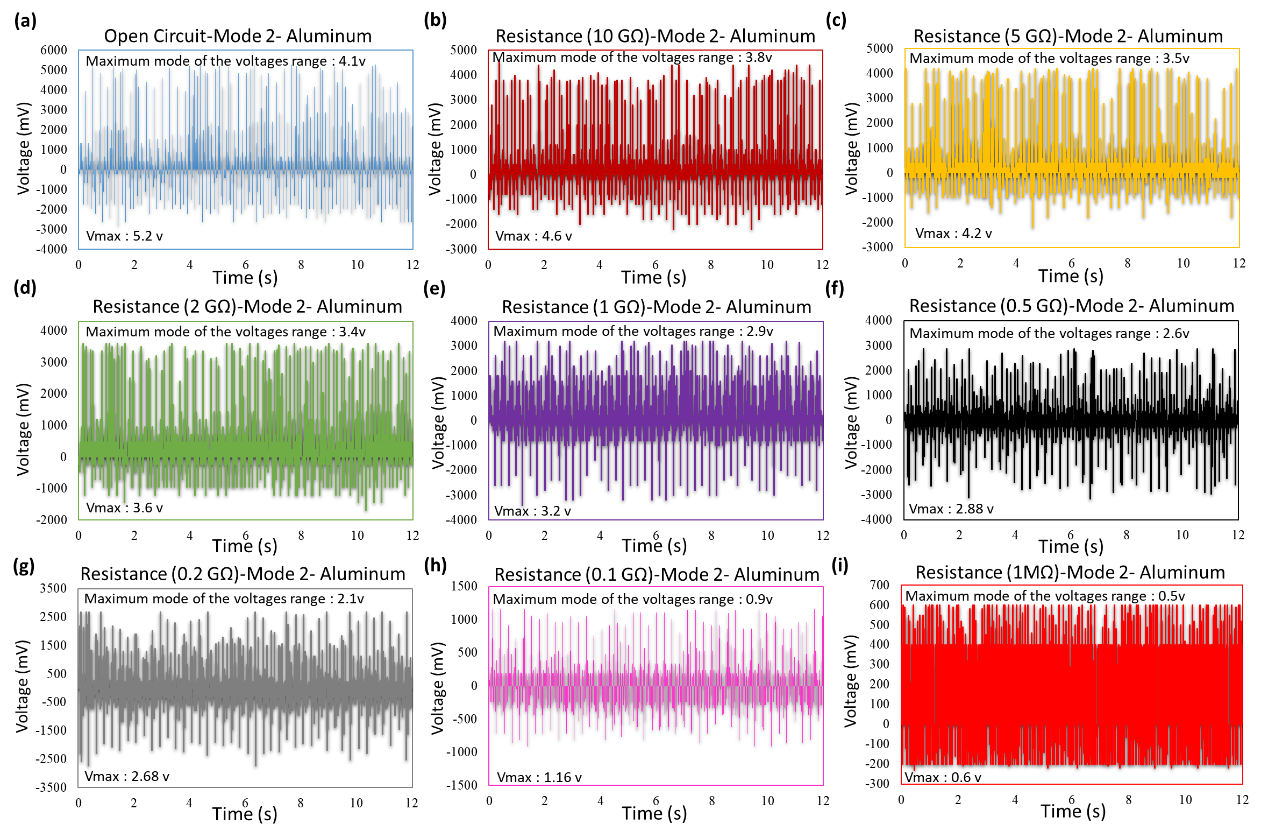


**Fig. S8.** **Energy harvesting signals of the Aluminum MC-TENG in Mode 2.** (a) Voltage distribution of copper in open circuit. (b) Voltage distribution of copper with the electrical resistance 10ΩG, (c) 5 ΩG, (d) 2 ΩG, (e) 1 ΩG, (f) 0.5 ΩG, (g) 0.2 ΩG, (h) 0.1 ΩG, (i) 1MG.


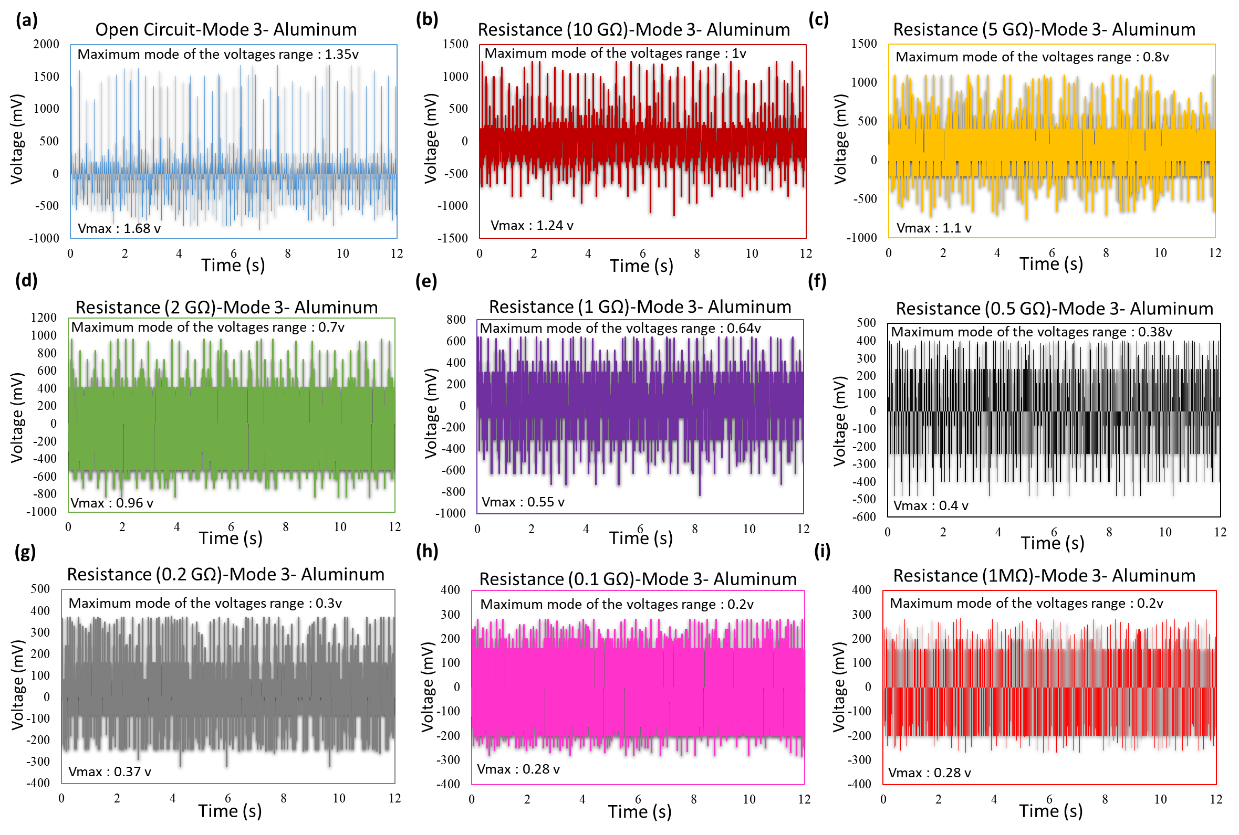


**Fig. S9.** **Energy harvesting signals of the Aluminum MC-TENG in Mode 3.** (a) Voltage distribution of copper in open circuit. (b) Voltage distribution of copper with the electrical resistance 10ΩG, (c) 5 ΩG, (d) 2 ΩG, (e) 1 ΩG, (f) 0.5 ΩG, (g) 0.2 ΩG, (h) 0.1 ΩG, (i) 1MG.

**5. Supplemental Videos**

The Supplemental Videos present the experimental response and results of the MC-TENG. Supplemental Video 1 presents the oscillation scenarios of the capsulate TENG. Supplemental Video 2 presents the response of the copper MC-TENG in mode 2.
